# Discovery of Dihydrochalcone as Potential Lead for Alzheimer’s Disease: *In Silico* and *In Vitro* Study

**DOI:** 10.1371/journal.pone.0079151

**Published:** 2013-11-18

**Authors:** Man Hoang Viet, Chun-Yu Chen, Chin-Kun Hu, Yun-Ru Chen, Mai Suan Li

**Affiliations:** 1 Institute of Physics, Polish Academy of Sciences, Warsaw, Poland; 2 Genomics Research Center, Academia Sinica, Taipei, Taiwan; 3 Institute of Physics, Academia Sinica, Nankang, Taipei, Taiwan; 4 Institute for Computational Science and Technology, Quang Trung Software City, Ho Chi Minh City, Vietnam; Russian Academy of Sciences, Institute for Biological Instrumentation, Russian Federation

## Abstract

By the virtual screening method we have screened out Dihydrochalcone as a top-lead for the Alzheimer’s disease using the database of about 32364 natural compounds. The binding affinity of this ligand to amyloid beta (A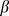) fibril has been thoroughly studied by computer simulation and experiment. Using the Thioflavin T (ThT) assay we have obtained the inhibition constant IC50 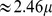
M. This result is in good agreement with the estimation of the binding free energy obtained by the molecular mechanic-Poisson Boltzmann surface area method and all-atom simulation with the force field CHARMM 27 and water model TIP3P. Cell viability assays indicated that Dihydrochalcone could effectively reduce the cytotoxicity induced by A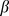. Thus, both *in silico* and *in vitro* studies show that Dihydrochalcone is a potential drug for the Alzheimers disease.

## Introduction

Alzheimer’s disease (AD) is the most common form of dementia among the senior population that is increasing substantially as populations age [1]. The patient with AD will lose memory, decay language, and experience problems with visual spatial search *etc*. AD may be pathologically characterized by progressive intracerebral accumulation of beta amyloid (A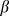) peptides [2] and tau protein [3]. However, genetic and pathological evidences strongly support the first hypothesis [4]. The A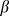 peptides are proteolytic by-products of the amyloid precursor protein and are most commonly composed of 40 (A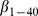) and 42 (A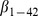) amino acids. A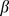 peptides appear to be unstructured in monomer state but aggregate to form fibrils with an ordered cross-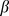-sheet pattern [5], [6]. Increasing evidence from recent studies indicates that both soluble oligomers and mature fibrils are the toxic agents [2].

Presently, there is no cure or treatment for AD, and significant effort has, therefore, been made to find efficient drugs to cope with it. One of the promising approaches is to inhibit misfolding and reverse aggregation of amyloid peptides [7]. A large number of potential A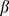 fibrillogenesis inhibitors have been proposed including polyamines, metal chelators, carbohydrate-containing compounds, polyphenols, osmolytes, short peptides, and RNA aptamers etc [7], [8]. One should mention a number of small molecule inhibitors such as poly-L-lysine [9], dopamine [10], L-dopa [11], melatonin [12], indole-3-propionic acids [13], apomorphine derivatives [14], salvianolic acids [15] and many others. Nutraceuticals, which are natural products or extracts therefrom, as shown by preclinical and certain clinical studies, might be of value as AD therapeutic agents [7].

In this paper we carry out the comprehensive study of binding affinity of compounds derived from Eastern herbs and plants to aggregates of A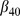 and A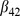. Having used the docking method we screened out 20 top-leads for 6A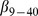 and 5A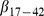 fibrils. However, we were able to purchase only compound Dihydrochalcone which is an extract from Daemonorops draco tree. Our experimental study shows that this compound is promising for AD having the inhibition constant IC50 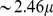
M and low toxicity. The experimental result of the inhibition constant has been confirmed by the molecular mechanic-Poisson Boltzmann surface area (MM-PBSA) method which is more sophisticated than the docking method. Thus for the first time by the experiment and simulation we predict that Dihydrochalcone is a good candidate for AD.

## Materials and Methods

### Data Base of Compounds

We consider 32364 compounds derived from Eastern herbs and plants (see website: http://tcm.cmu.edu.tw). Applying Lipinski’s rule of five [16] (see File S1) to this data base we obtained 3699 ligands that have drug-like properties and satisfy the Lipinski’s rule. However, one should bear in mind that many natural products remain bioavailable despite violating the Rule of Five [17], [18]. The virtual screening was applied to the reduced set of compounds.

### Receptors

In order to study the binding affinity to mature fibrils we choose two typical structures of 6A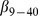 obtained from Prof. R. Tycko [19] and 5A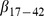 (PDB ID: 2BEG [6]). Note that 8 and 16 disordered residues of the N-terminal of A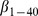 and A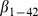 are neglected from fibril constructions. In the case of A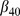, several NMR fibril structures are available, but to make a reasonable comparison between two types of fibrils, we choose 6A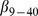 because its structure is closest to 2BEG.

### Docking Method

Both ligand-based and structure-based virtual screening methods have their advantages and drawbacks. However, since the docking methods are able to select more diverse actives than ligand-based methods like 2D similarity or substructure searching [20], we will use Autodock Vina method [21]. In our simulations the docking score is the binding energy and the best docking mode is the highest scoring pose or the conformation with the lowest binding energy. The details of this method are available in File S1.

### Molecular Dynamics Simulation

In order to estimate the binding free energy of Dihydrochalcone to 6A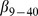 by the MM-PBSA method we have carried the molecular dynamics simulation using the force field CHARMM 27 [22] and water model TIP3P [23]. More details are described in File S1.

### MM-PBSA Method

We have applied the MM-PBSA method to estimate the binding free energy of Dihydrochalcone to 6A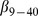. The details of this method are given in our previous works [24]. Overall, in the MM-PBSA approach the binding free energy of ligand to receptor is defined as follows
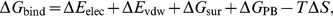
(1)where 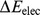 and 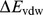 are contributions from electrostatic and vdW interactions, respectively. 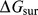 and 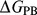 are nonpolar and polar solvation energies. The entropic contribution 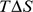 is estimated using the normal mode approximation. In order to calculate 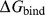, the molecular dynamics (MD) simulations have been carried out using the force field CHARMM 27 [22] and water model TIP3P [23]. The structures of receptor–ligand complex obtained in the best docking mode are used as starting configurations for simulations. For the 6A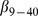-Dihydrochalcone complex four 20 ns MD trajectories were generated. Snapshots collected in equilibrium are used to compute the binding free energy given by Eq. 1.

### A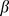 Preparation

To prepare the A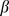 stock, lyophilized A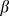 40 peptide, 0.5 mg, was freshly dissolved in 145 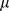
l Buffer A (10 mM Tris-HCl, pH = 7.4, and 150 mM NaCl) containing 8 M GdnHCl and refolded into Buffer A at a concentration of ∼1 mg/ml. Then, the stock was centrifuged at 17,000 × g, 4°C for 30 min. The supernatant was collected and quantified by absorbance at 280 nm (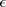 = 1,280 cm
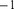
M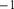) and used as a stock solution to prepare A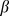 at 25 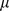
M for all experiments [25], [26].

### Dihydrochalcone Preparation

Dihydrochalcone was purchased from MP Biomedicals, Inc. (Fountain Pkwy, OH). Stock of Dihydrochalcone, 20 mM, was dissolved in 100% DMSO and diluted to different concentrations as indicated. The final DMSO concentration was constant in each condition.

### Thioflavin T (ThT) Assay

A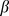 (25 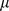
M) in Buffer A with different Dihydrochalcone concentrations and 25 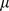
M ThT were incubated in a 384-well ELISA plate and monitored by a microplate reader (SpectraMax M5; Molecule Devices) at 25°C. The samples were constantly rotated at 400 rpm during the incubation. The ThT fluorescence was measured at 485 nm where the excitation was at 442 nm. The final ThT intensity was plotted against the compound concentration and fitted to obtain IC50 using the equation, Y = 100/(1+10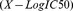) in *Prism 5* (GraphPad Software, Inc, Avenida de la Playa, CA).

### TEM

The aggregated samples were placed on glow-discharged, 400-mesh Formvar carbon-coated copper grids (EMS Inc., Hatfield, PA, USA) for 3 min, rinsed, and negatively stained with 2% uranyl acetate. The samples were examined with a Hitachi H-7000 TEM (Hitachi Inc., Tokyo, Japan) with an accelerating voltage of 75 kV.

### MTT Assay

The HEK293 cells were seeded into 96-well plates (100 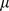
l/well) one day prior to the experiment. The cells were treated with the end-point products and incubated at 37°C for 24 hr. MTT solution (Sigma) was then added to each well and incubated for another 4 hr. The medium was removed and 100 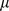
l of DMSO was added to dissolve the formazen crystals. The absorbance (A) was measured at 570 nm and the background signals caused by the samples without cells were subtracted. The data were normalized using the buffer control as 100%.

## Results and Discussion

### Theoretical Results

#### Top leads revealed by the virtual screening

The positions of 3699 ligands in the best docking mode for two targets are shown in Fig. S1 in File S1. In the case of 6A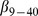 all compounds are positioned inside the fibril and mainly near to the loop region. Most of them have contacts with peptides II – V. Only few ligands are located near terminals of peptides. The situation is very different in the case of 17A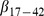, where binding sites are scattered not only inside but also outside of fibrils.

As follows from the distributions of binding energies obtained in the best docking mode (Fig. S2 in in File S1), ligands show higher binding affinity toward 6A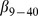 compared to 5A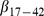. This is presumably because they are mainly located outside 5A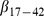. The most probable energies are about −6 and −8 kcal/mol for 5A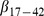 and 6A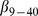, respectively.

**Figure 1:**
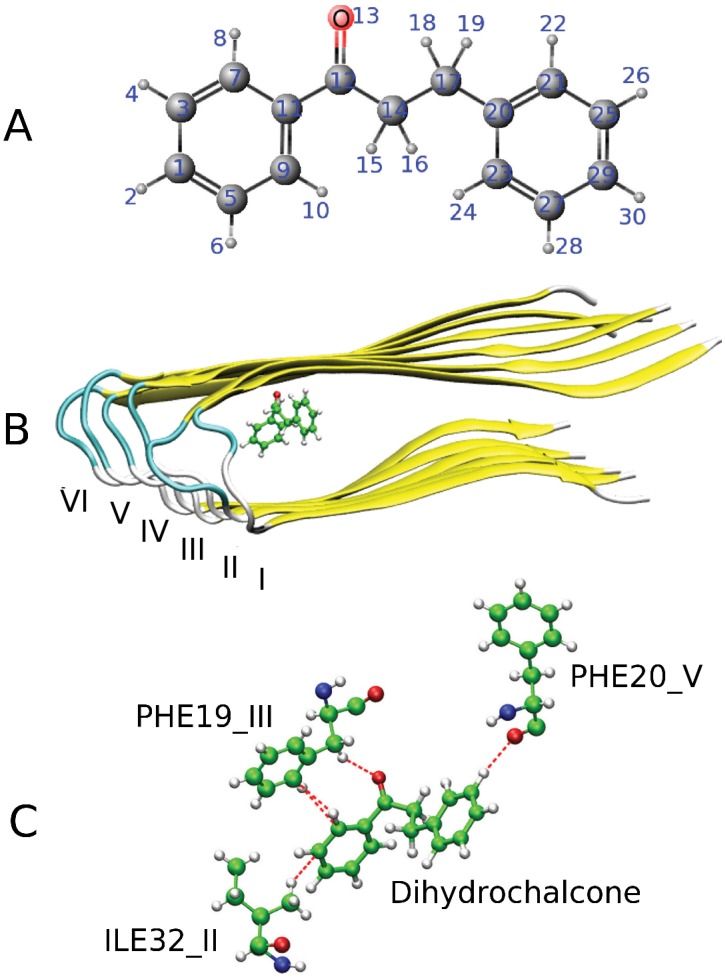
Chemical structure and the best docking pose of Dihydrochalcone. (A) Structure of Dihydrochalcone. (B) The best docking conformation of the 6A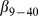-Dihydrochalcone complex. (C) Hydrogen bonds between Dihydrochalcone and fibril 6A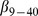 in the best docking mode. The ligand has 1, 3 and 1 hydrogen bonds with residues ILE32 of chain II, PHE19 of chain III and PHE20 of chain V, respectively.

We have made a ranking of ligands by their binding energies to two receptors. The 10 top leads are listed on Table S1 in File S1. Dia-aurantiamide acetate (ID: 30140) is a champion with 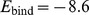 kcal/mol to 5A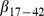. In the case of 6A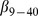 Delavinone (ID: 32022) has the lowest binding energy. The common feature of 10 top leads is that they contain at least two rings which favor high binding affinity. Among them Dihydrochalcone is the lightest compound having weight of 200 Da. Moreover, the structure of this compound is similar (using the software SHAEP software (http://users.abo.fi/mivainio/shaep/) [27] one can show that the shape similarity between Dihydroachalcone and Curcumin is 67.12%) to that of curcumin undergoing the second phase of clinical trials. Both of them have two aromatic rings (Fig. 1A and Table S1 in File S1), which, as shown below, play a decisive role in binding affinity. From 10 top leads (Table S1 in File S1) we were able to purchase Dihydrochalcone, which is derived from Daemonorops draco tree (Fig. S3 in File S1), to perform *in vitro* study for its ability to prevent A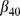 aggregation. Therefore we consider this compound in more detail.

#### Hydrogen network of dihydrochalcone

The 6A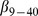-Dihydrochalcone complex in the best docking mode (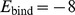 kcal/mol) is shown in Fig. 1B. In this configuration Dihydrochalcone forms 1, 3 and 1 hydrogen bonds (HB) with residues ILE32 of chain II, PHE19 of chain III and PHE20 of chain V, respectively (Fig. 1C). Note that among all of five non-standard HBs one has three C-H…C and two C-H…O bonds that may be important for the interaction of small molecules with other molecules [28], [29]. Fig. S4 (File S1) shows the dependence of binding energies of 3699 ligands to target 6A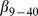 on the number of HBs. Since the correlation between two these quantities is very low one can conclude that the HB network is not strong enough to play the key role in the binding affinity to A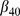 fibrils. This conclusion is also valid for A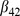 target (results not shown).

#### Estimation of binding free energy of dihydrochalcone to 6A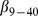 by MM-PBSA method

It is well known that the docking method is not accurate enough due to omission of ligand dynamics and a limited number of trial positions of ligand. Therefore we will use the MM-PBSA method which is more reliable in estimating the binding free energy 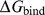 (Eq. (1)).

We use the conformation obtained in the best docking mode (Fig. 1B) as a starting conformation for MD simulation. Four independent 20 ns MD trajectories have been generated with different random seed numbers that are needed to create different pools for initial velocities of atoms. The time dependence of C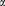 root mean square displacement (RMSD) of the receptor 6A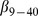 shows that the systems reaches equilibrium (curves reach saturation) after about 10 ns (Fig. S5 in File S1).

We have stored snapshots every 10 ps during last 10 ns for MM-PBSA calculation using Eq. (1). Although 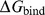 is sensitive to MD runs (Table S2 in File S1) we have the clear trend that the van der Waals interaction dominates over the electrostatic interaction. The entropic and nonpolar (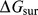) contributions are almost homogeneous over for all trajectories. Averaging over four MD runs we obtain 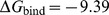 kcal/mol which corresponds to the inhibition constant IC50 ∼1 *μ*M. This result is in reasonable agreement with our experimental data (see below).

We have aslo studied the binding 3 ligands Delavinone, Sisalagenin and Sipeimine that can cross the blood brain barrier easily (log(BB)>0, see below) using the MM-PBSA method. As in the case of Dihydrochalcone the systems reach equilibrium after about 10 ns (Fig. S5 in File S1). Within the error bars they have the binding free energy compatible to Dihydrochalcone (Table S2 in File S1). Therefore. these compounds also deserve further *in vitro* and *in vivo* studies.

#### Aromatic rings play the key role in ligand binding

We have considered the contributions of individual atoms of Dihydrochalcone to the electrostatic and van der Waals interactions with the receptor (Fig. S6 in File S1). The results have been obtained as averages over four MD trajectories. Except atom 11 (Fig. 1A) all Carbon atoms have the repulsive interaction with the receptor, while the Coulomb interaction with hydrogen atoms is attractive. The elctrostatic interactions of aromatic rings are almost compensated (Fig. S6 in File S1). The electrostatic interaction between Oxygen atom with the receptor is the strongest one. The contribution of Carbon atoms 12 and 17 from the middle part (Fig. 1A) is more important than Carbon atom 14. Similar to Curcumin case [24], two aromatic rings are very important in binding affinity of Dihydrochalcone to fibril.

#### Ligand binding slows down the fibril growth process

In this section we discuss the impact of ligand binding on fibril assembly and disassembly at the qualitative level. As evident from Fig. 1, the ligand is located inside fibril leading to its stabilization. The fact that the ligand binding can slow down fibril assembly may be qualitatively understood as follows. Suppose that the fibril growth proceeds by addition of a nascent monomer to the preformed template [30], [31]. Then the fluctuations of template would facilitate this process [31]. From this point of view the stabilization by ligand binding makes the template more rigid reducing the fibril formation rates as observed in our ThT fluorescence experiments.

The question about the influence of ligand binding on fibril disassembly looks more complicated. In our opinion, there is a possibility that ligands locate not only inside but also outside fibrils. Then outside ligands may destabilize fibrils, but this question requires further investigation.

#### Blood-Brain Barrier (BBB)

The BBB is a physical barrier in the circulatory system that a compound should across in order to travel into the central nervous area [32]. Thus the requirement of passing this barrier is necessary for any AD drug candidate. The crossing ability is measured by log(BB) which is the logarithm base 10 of the ratio of the compound concentration in the brain to that in the blood. Using the quantitative structure-activity relationship (QSAR) implemented in the PreADME prediction sofware [33] we obtained log(BB) = 0.18 for Dihydrochalcone. This implies that the crossing ability of Dihydrochalcone is much better than Curcumin which has log(BB) = −1.04.

## Experimental Results

**Figure 2:**
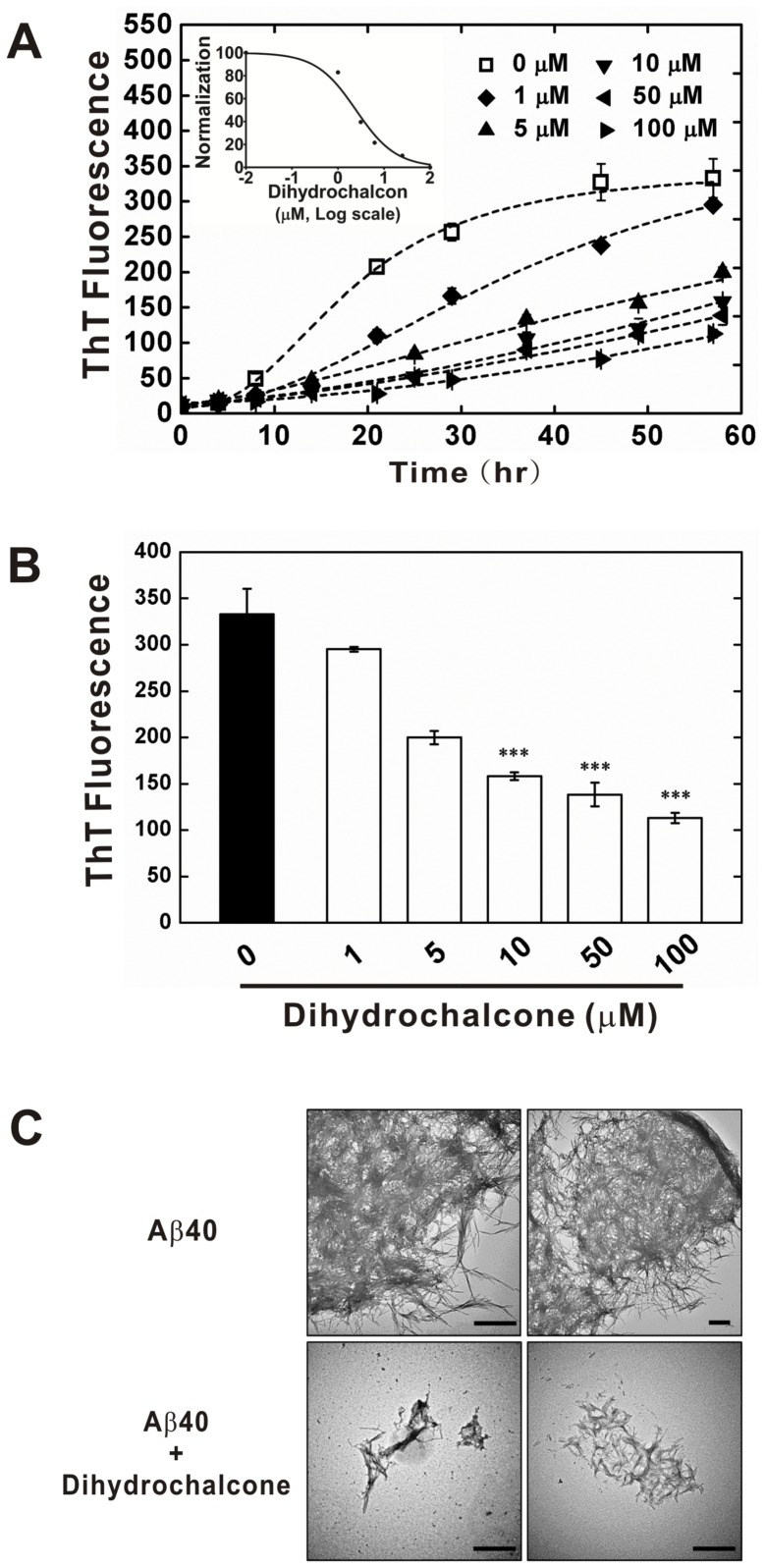
Dihydrochalcone suppresses A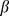 fibrillization. (A) A
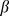
40 (25 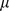
M) was incubated at 25°C with different concentrations of Dihydrochalcone in the presence of ThT and the fibrillization was monitored by ThT fluorescence for 58 hr. A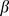 in the absence (0 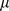
M, 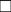) and presence of various concentrations of Dihydrochalcone (1 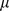
M, ♦; 5 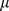
M, ▴; 10 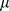
M, ▾; 50 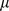
M, ◂; 100 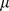
M, ▸). The final ThT intensity was plotted against Dihydrochalcone concentrations and shown in the inset (IC50 = 2.46 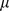
M) of panels (A) and (B).The mean value of the final ThT intensity and statistical significance was shown in panel (B). One-way ANOVAs, ***, P<0.0001. (C) TEM images of the end-point products from ThT experiments. Scale bars = 500 nm.

**Figure 3:**
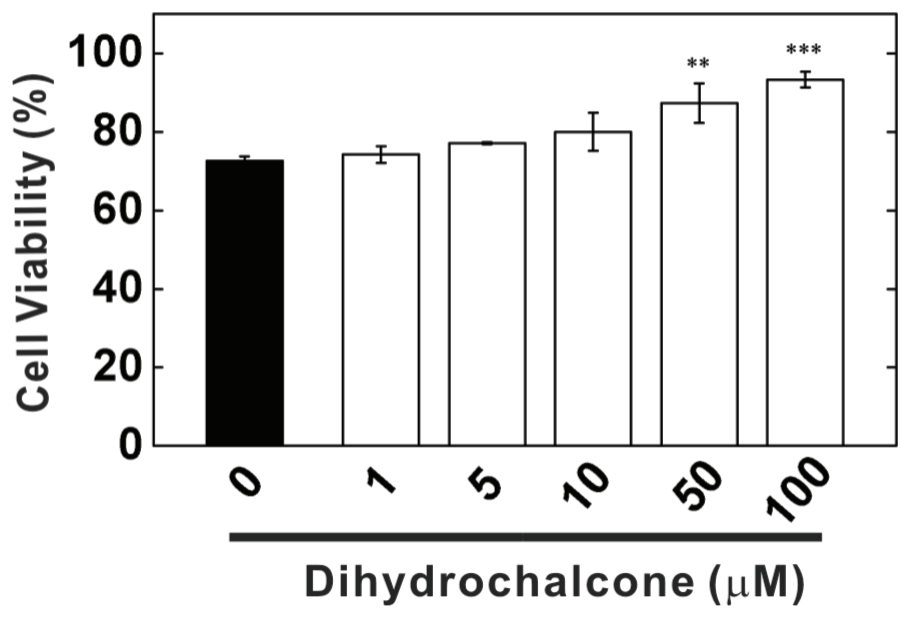
Cytotoxicity of the end-point products of A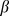 fibrillization with and without Dihydrochalcone. HEK293 cells were treated with the end-point products of A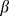 fibrillization with and without various concentrations of dihydro-chalcone as indicted in Figure 2 for 24 hr and subjected to MTT assay. Triplicate experiments were performed and the data were shown as mean ± standard deviation. The statistical significance was indicated by one-way ANOVAs (**, P<0.005; ***, P<0.0005).

To validate the potency of the natural compounds virtually screened from the data base, we performed the A
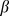
40 fibrillization experiment with Dihydrochalcone that is commercially available. We first employed Thioflavin T (ThT) assay to detect A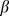 fibrillization in the presence of different concentrations of Dihydrochalcone. Five concentrations ranging from 1 to 100 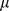
M of Dihydrochalcone were used to examine the inhibitory effect. In the absence of Dihydrochalcone, A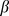 rapidly increased in 7 hr and reached a steady state after 30 hr monitored by ThT assay (Fig. 2A). After addition of 1 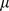
M Dihydrochalcone, the elongation phase of A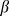 fibrillization was singnificantly decreased. Upon increasing Dihydrochalcone, the ThT intensity at the steady state decreased in a dose dependent manner. In the presence of 100 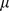
M Dihydrochalcone, ThT intensity decreased to 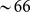
% of that in the absence of Dihydrochalcone suggesting reduction of A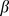 fibrils. We plotted the intensity versus Dihydrochalcone concentration and calculated its IC50 to be ∼2.46 *μ*M (Figure 2A, inlet), that is comparable with our theoretical estimation and with that of curcumin [34].The averaged final ThT intensity was plotted (Figure 2B). The data showed the inhibitive effect of Dihydro-chalcone was statistically significant. We also compared the inhibitive effect of Dihydrochalcone with the reported A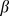 fibril inhibitors including curcumin, rosmarinic acid, and resveratrol (Figure S7 and S8 in File S1) by ThT assay. The IC50 of Dihydrochalcone was similar to those of curcumin and resveratrol but better than rosmarinic acid. To confirm the fibril quality and morphology, we used transmission electron microscopy (TEM) to examine the end-point products of the ThT experiments (Fig. 2C). In the absence of Dihydrochalcone, A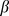 fibrillized to clusters of thread-like fibrils in high density. With higher concentration of Dihydrochalcone, we observed much fewer amyloid fibrils by TEM and the clusters were much reduced. We further examined the cytotoxicity of the end-point products by MTT assay using HEK293 cells (Fig. 3). In the absence of Dihydrochalcone, A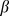 treated cells have 72.5% viability than the buffer control. The cytotoxicity was significantly reduced with higher Dihydrochalcone concentrations. The cell viability was increased to 93.2% in the presence of 100 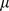
M Dihydrochalcone comparing to the buffer control. The result clearly showed that Dihydrochalcone can inhibit A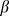 aggregation and toxicity.

## Conclusion

By virtual screening we have sorted out the most potent candidates for AD from the large data base of natural products. *In silico* and *in vitro* studies clearly show that the extract from Daemonorops draco tree Dihydrochalcone satisfies requirements for a AD drug such as the binding affinity, BBB crossing ability and non-toxicity. Therefore we recommend it for further *in vivo* study and possible clinical trials.

## Supporting Information

File S1 **Lipinski’s rule of five; docking method; molecular dynamics simulation and supporting tables and figures.**
(PDF)
