# Supplementary material for: Discovery of Dihydrochalcone as Potential Lead for Alzheimer’s Disease: In Silico and In Vitro Study
Source: PLoS One. 2013 Nov 18;8(11):e79151. doi: 10.1371/journal.pone.0079151 (PMC3832475; doi:10.1371/journal.pone.0079151)
Supplement: File S1 — Lipinski’s rule of five; docking method; molecular dynamics simulation and supporting tables and figures. (PDF) [file pone.0079151.s001.pdf]

Supporting Information

Filtering database by Lipinski's rule of five.

In order to narrow down the number of compounds we use the Lipinski's rule of five [1] which states that a compound is more likely to be membrane permeable and easily absorbed by the body if it matches the following criteria:

- The molecular weight is less than 500 daltons.
- The octanol-water partition coefficient logP not greater than 5.
- Not more than 5 hydrogen bond donors (nitrogen or oxygen atoms with one or more hydrogen atoms).
- Not more than 10 hydrogen bond acceptors (nitrogen or oxygen atoms)

Applying these factors to the Eastern herb database [2] we have reduced the number of compounds from 32364 to 3699 which will be further studied by the docking method. The calculation has been done by the Calculator Plugins of Chemaxon package [3].

Docking method

AutodockTools 1.5.4 [4] is used to prepare PDBQT files for the full-length Aβ peptide, fibrils of truncated peptides and three ligands. To dock compounds from the reduced set to receptors PDBQT files were used as the input for the Autodock Vina version 1.1 [5] which uses the idea of empirical scoring [6] that the total binding free energy can be separated into several physically distinct contributions. Autodock Vina is approximately two orders of magnitude faster than Autodock 4 [5]. Moreover this method also significantly improves the accuracy of the binding mode prediction compared with Autodock 4 [5].

A modified version of the CHARMM force field was implemented [7,8] to describe atomic interactions. In Autodock Vina the Broyden Fletcher Goldfarb Shanno method [9] is employed for local optimization. To obtain accurate results we set the exhaustiveness of global search equal 300. The maximum energy difference between the worst and best binding modes was chosen to be 7. Twenty binding modes (20 modes of docking) were generated with random starting positions of the ligand, which has fully flexible torsion degrees of freedom. The receptor flexibility is not allowed in our simulations. The center of grids was placed at the center of mass of the receptor. Grid dimensions were chosen as 80×50×65 and 75×55×65 for 6Aβ_{9-40} and 5Aβ_{17-42}, respectively. They are large enough to cover the entire receptor.

Molecular dynamics simulation

The best docking mode of 6Aβ_{9-40}-Dihydrochalcone complex has been used as initial configuration for all-atom MD simulation using the force field CHARMM 27 [10] and water model TIP3P [11]. All calculations were carried with the help of Gromacs-4.5 package [12]. The complex is placed in a triclinic box of around 19000 water molecules with 1nm distance between the solute and box. Periodic boundary condition is imposed with 1.4 nm and 1.0 nm cut-off for van der Waals and electrostatic interactions, respectively. Long range electrostatic interactions were computed by particle-mesh Ewald summation method [13]. Equations of motion were integrated using a leap-frog algorithm [14] with a time step 1 fs. The non-bonded interaction pair-list was updated every 10 fs with the cut-off of 1 nm. All systems were neutralized by adding counter-ions and then minimized to remove the local strain in the protein while adding full hydrogens and to remove bad Van der Waals contacts with waters. By adding the conjugate gradient method for every 50 steps of steepest descent, minimization is converged when maximum force decreases to smaller than 0.01 kJ/mol/nm. Then, atoms of protein were restrained, leaving the remains

to be relaxed for 100 ps in order to obtain evenly distributed systems. The temperature was gradually heated to 300 K during 100 ps with 5 kcal/mol harmonic restraints in all systems. The equilibration was next performed, coupling with temperature and pressure. Constant temperature at 300 K was enforced using Berendsen algorithm [15] under 50 ps NVT simulation with a damping coefficient of 0.1 ps. The Parrinello-Rahman pressure coupling [16] was used in 100 ps NPT run for 1 atm constant atmosphere with damping coefficient of 0.5 ps. Final NPT simulations of 20 ns were carried out with 1 fs time step.

References

1. Lipinski A, Lombardo F, Dominy BW, Feeney PJ (1997) Experimental and computational approaches to estimate solubility and permeability in drug discovery and development settings. Adv Drug Del Rev 23: 3-25.

2. Chen CYC (2011) Tcm database@taiwan: The world's largest traditional chinese medicine database for drug screening *in silico*. Plos One 6: e15939.

3. ChemAxon (2011). Calculator plugins were used for structure property prediction and calculation, marvin 5.6.0.2, chemaxon. `http://www.chemaxon.com`.

4. Sanner MF (1999) Python: A programming language for software intergration and development. J Mol Graphics Mod 17: 57-61.

5. Trott O, Olson AJ (2010) Improving the speed and accuracy of docking with a new scoring function, efficient optimization, and multithreading. J Comput Chem 31: 455-461.

6. Bohm H (1994) The development of a simple empirical scoring function to estimate the binding constant for a protein-ligand complex of known three-dimensional structure. J Comput Aided Mol Design 8: 243–256.

7. Morris GM, Godsell DS, Halliday RS, Huey R, Hart WE, et al. (1998) Automated docking using a lamarckian genetic algorithm and an empirical binding free energy function. J Comput Chem 19: 1639-1662.

8. Morris GM, Goodsell DS, Huey R, Olson AJ (1996) Distributed automated docking of flexible ligands to proteins: Parallel applications of autodock 2.4. J Comput-Aided Mol Des 10: 293-304.

9. Shanno DF (1970) Conditioning of quasi-newton methods for function minimization. Matthematics of Computation 24: 647-656.

10. Brooks BR, III CLB, MacKerell JAD, Nilsson L, Petrella RJ, et al. (2009) Charmm: The biomolecular simulation program. J Comp Chem 30: 1545–1614.

11. Jorgensen WL, Chandrasekhar J, Madura JD, Impey RW, Klein ML (1983) Comparison of simple potential functions for simulating liquid water. J Chem Phys 79: 926–935.

12. Hess B, Kutzner C, van der Spoel D, Lindahl E (2008) Gromacs 4: Algorithms for highly efficient, load-balanced, and scalable molecular simulation. J Chem Theor Comp 4: 435–447.

13. Darden T, York D, Pedersen L (1993) Particle mesh Ewald: An nlog(n) method for Ewald sums in large systems. J Chem Phys 98: 10089–10092.

14. Hockney RW, Goel SP, Eastwood J (1974) Quit high resolution computer models of plasma. J Comp Phys 14: 148-158.

15. Berendsen HJC, Postma JPM, Vangunsteren WF, Dinola A, Haak JR (1984) Molecular-dynamics with coupling to an external bath. J Chem Phys 81: 3684-3690.

16. Parrinello M, Rahman A (1981) Polymorphic transitions in single crystals: A new molecular dynamics method. J Appl Phys 52: 7182–7190.

No.	ID	formula	2D-structure	Name	Source	ΔE_{b1}	ΔE_{b2}	log(BB)
1	30140	$C_{27}H_{28}N_2O_4$	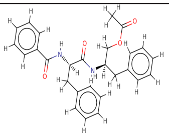	Diaauran -tiamide acetate	Artemisia anomala	-8.6	-9.4	-0.58
2	30682	$C_{17}H_{14}O_3$	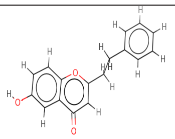	6-hydroxy-2-(2-phen -ylethyl) chromone	Aquilaria sinensis	-8.5	-9.0	-0.16
3	31005	$C_{17}H_{14}O_3$	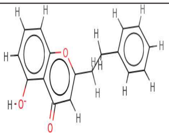	5-hydroxy-2-(2-phen -ylethyl) chromone	Sanguisorba officinalis	-8.5	-8.8	-0.09
4	**31867**	$C_{15}H_{14}O$	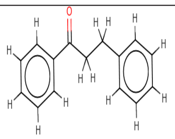	**Dihydroc - halcone**	**Daemonorops draco**	-8.5	-8.0	**0.18**
5	30678	$C_{18}H_{16}O_4$	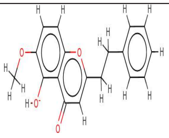	5-hydroxy-6-methoxy-2-(2-phen -ylethyl) chromone	Aquilaria sinensis	-8.4	-8.7	-0.69
6	32022	$C_{27}H_{43}NO_2$	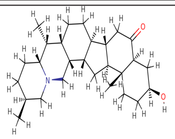	Delavinone	Fritillaria przewalskii Maxim. ex Batal	-7.1	-12.4	**0.75**
7	29443	$C_{27}H_{42}O_4$	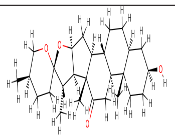	Sisalagenin	Agave sisalana Perrine	-7.7	-12.1	**0.26**
8	32101	$C_{27}H_{43}NO_3$	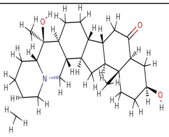	Sipeimine	Fritillaria pallidiflora Schrenk	-7.3	-11.6	**0.32**
9	29795	$C_{28}H_{40}O_6$	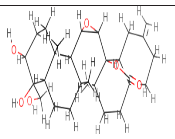	paeonenoide, a	Paeonia veitchii	-7.8	-11.3	-0.92
10	32256	$C_{22}H_{27}NO_5$	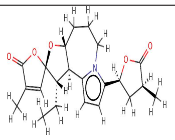	bisdehydro - stemoninine	Stemona sessilifolia	-6.2	-10.8	-0.48

Table S1. Top 10 leads, screened out from Eastern herbs and plants, by the Autodock vina method. Top binding energies to 5Aβ_{17-42} (ΔE_{b1}) and 6Aβ_{09-40} (ΔE_{b2}) (in kcal/mol) are marked in blue color.

	ΔE_{vdw}	ΔG_{sur}	ΔE_{ele}	ΔG_{PB}	$-T\Delta S$	$\Delta G_{binding}$
Dihydrochalcone	-25.6±0.7	-4.6±0.1	-4.9±3.9	16.2±5.4	9.5±0.5	-9.4±2.1
Delavinone	-49.7±1.0	-6.8±0.1	-2.5±3.2	30.2±2.5	15.9±0.3	-12.9±2.0
Sisalagenin	-45.6±1.0	-6.9±0.2	-4.3±3.1	29.4±3.1	15.5±0.3	-11.9±1.8
Sipeimine	-50.4±1.6	-7.1±0.3	1.5±2.1	28.8±4.2	15.5±0.4	-11.7±1.7

Table S2. Binding free energy of Dihydrochalcone, Delavinone, Sisalagenin and Sipeimine to 6Aβ_{09-40}. The result was obtained by the MM-PBSA method using the force field CHARMM 27 and water model TIP3P. The data is in kcal/mol and averaged over 4 MD trajectories.

‘

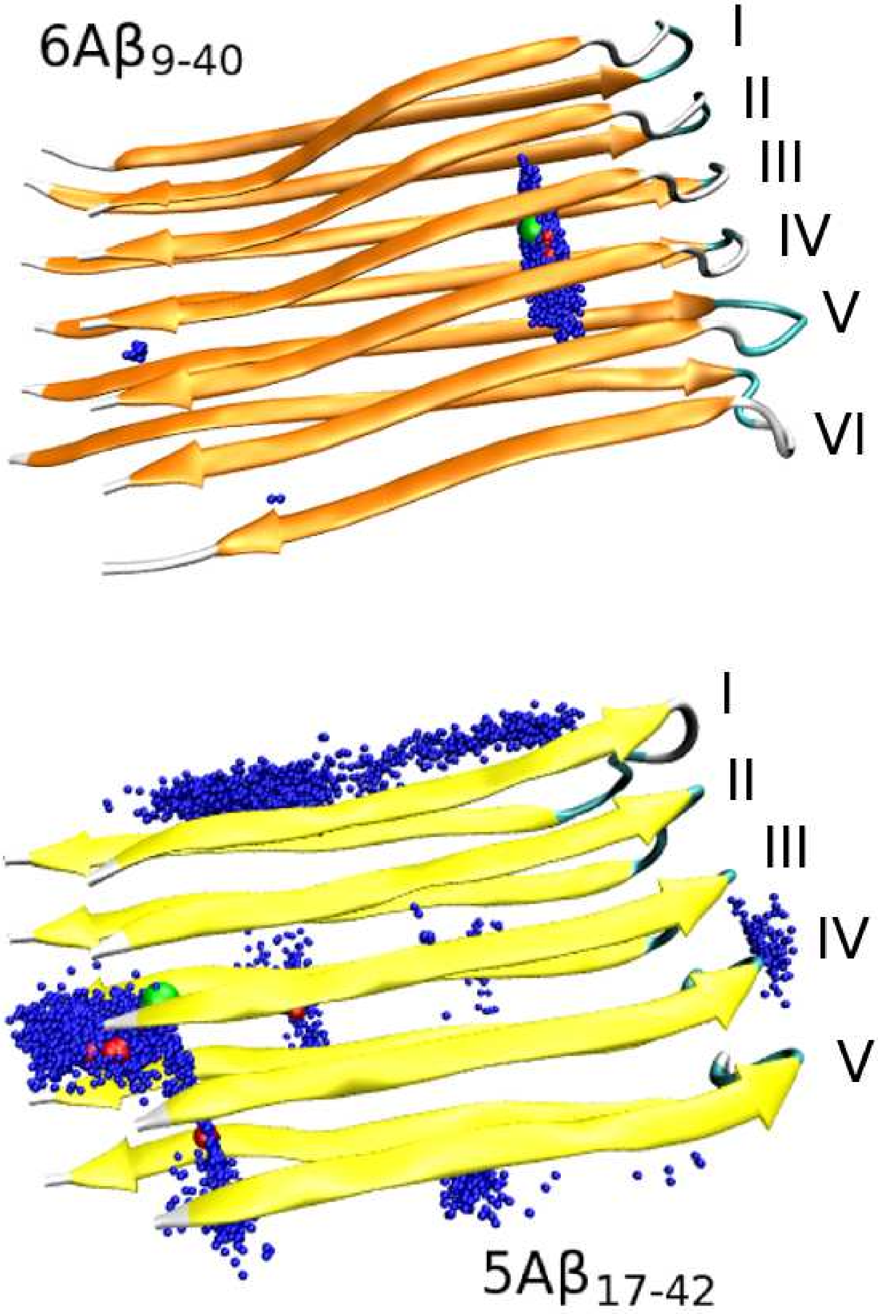

Figure S1. Binding positions of 3699 compounds (blues) from the traditional Chinese herbs to 6Aβ_{9-40} and 5β_{17-42}. Results have been obtained in the best binding mode. Red circles refer to representative top-hit binders, while green circle denotes Dihydrochalcone.

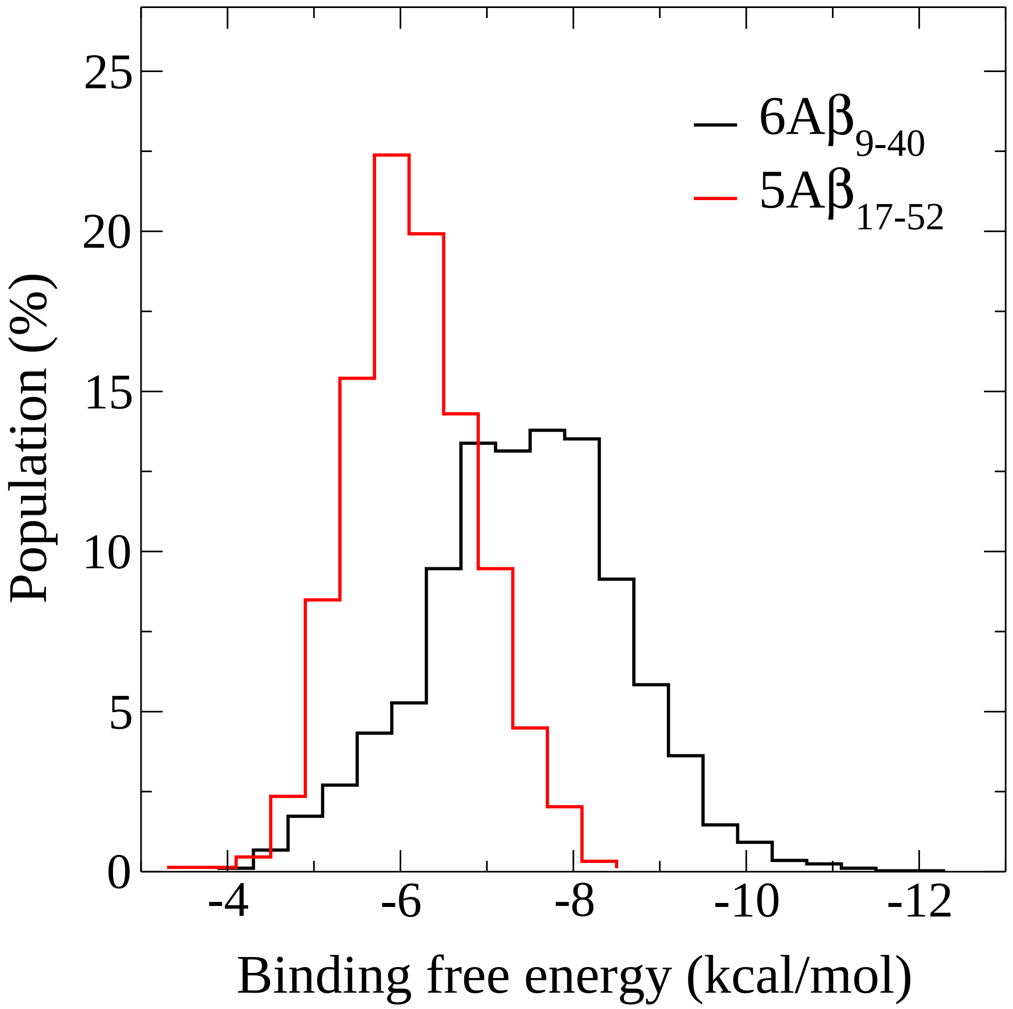

Figure S2. Distribution of binding energies obtained by the docking method. Population of binding energies of 3699 ligands to 6Aβ_{9-40} (black) and 5β_{17-42} (red). Results were obtained in the best docking mode.

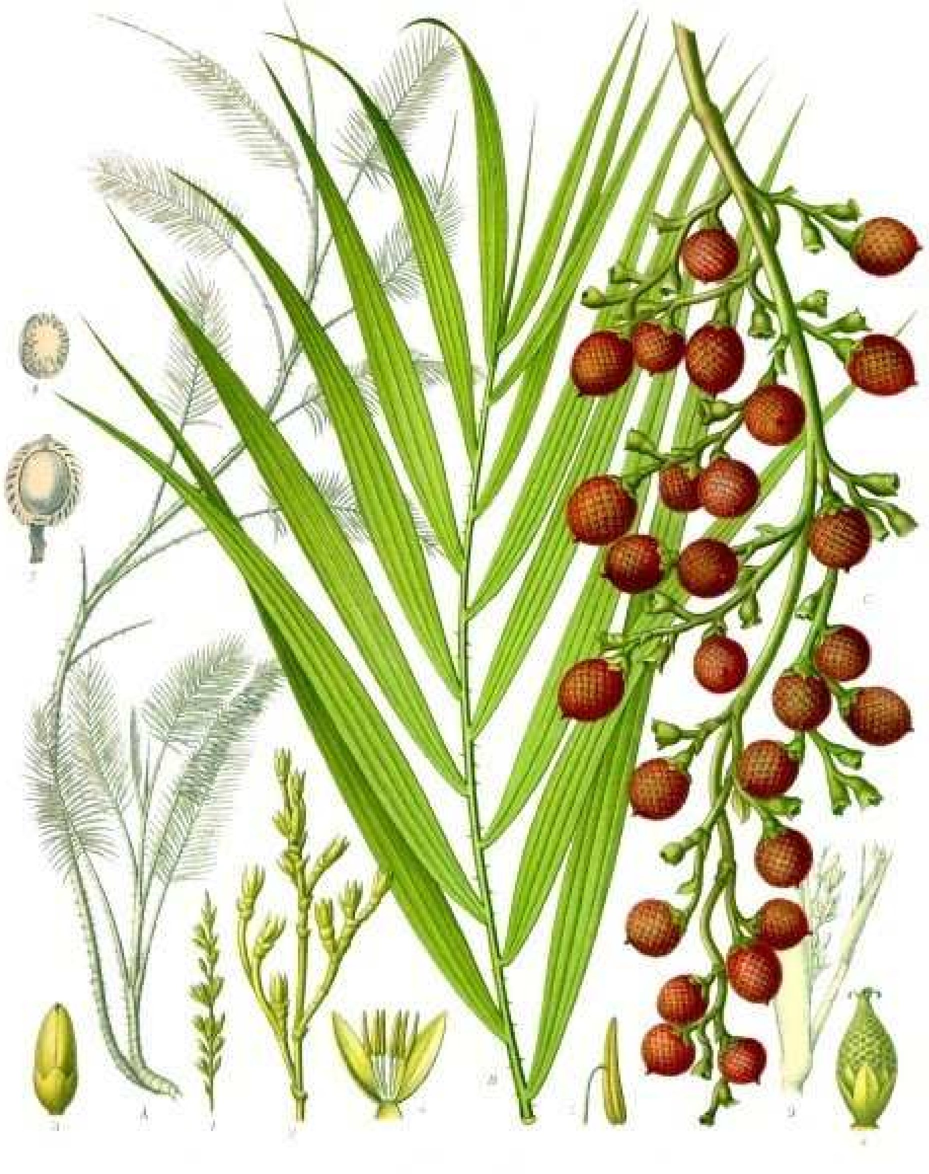

Figure S3. Daemonorops draco tree from which Dihydrochalcone is derived. Taken from en.wikipedia.org.

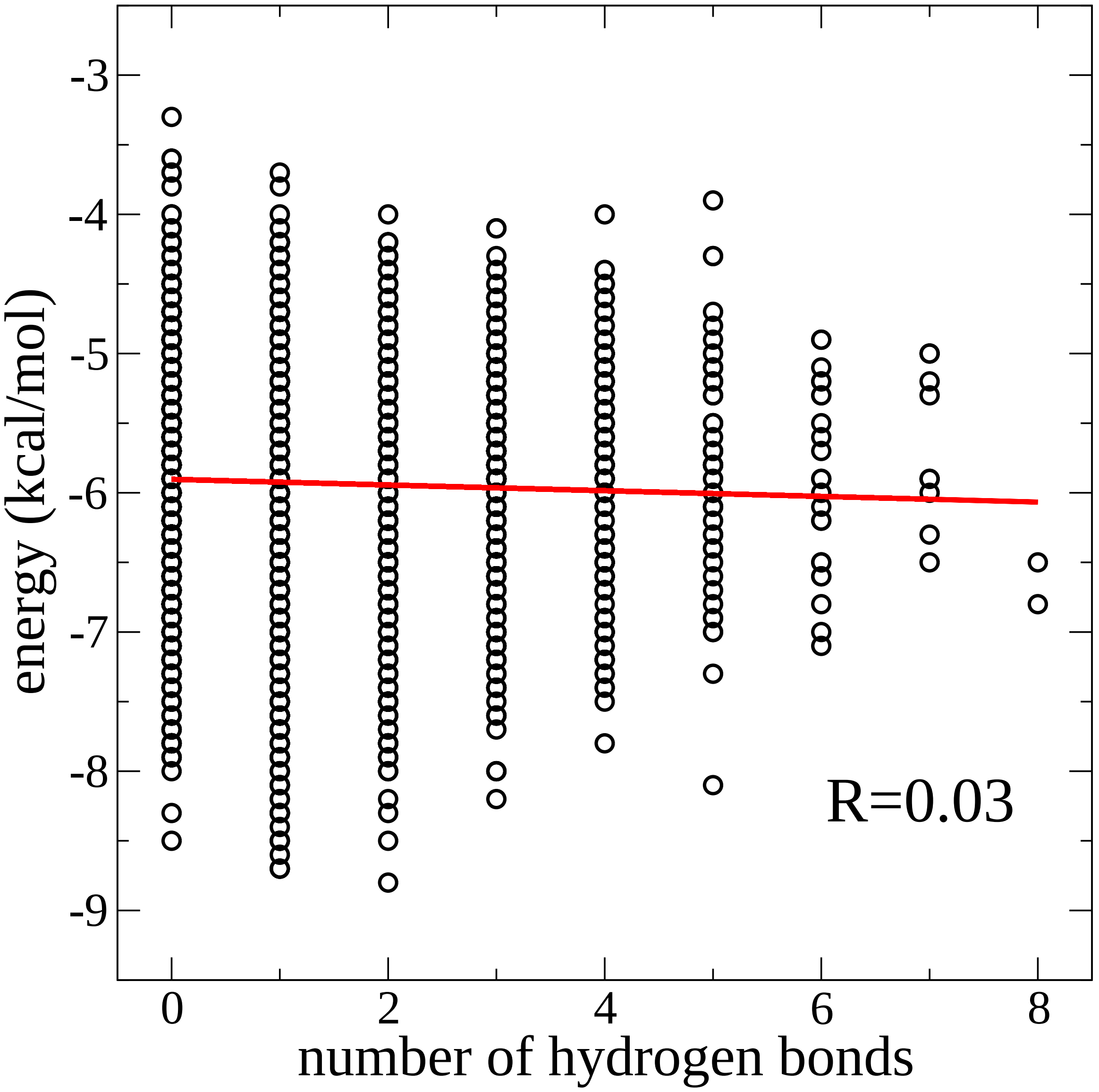

Figure S4. The dependence of the binding energies obtained in the best docking pose on the number of HBs. The correlation level $R \approx 0.03$. The results were obtained for 3699 ligands and target 6Aβ_{9-40}.

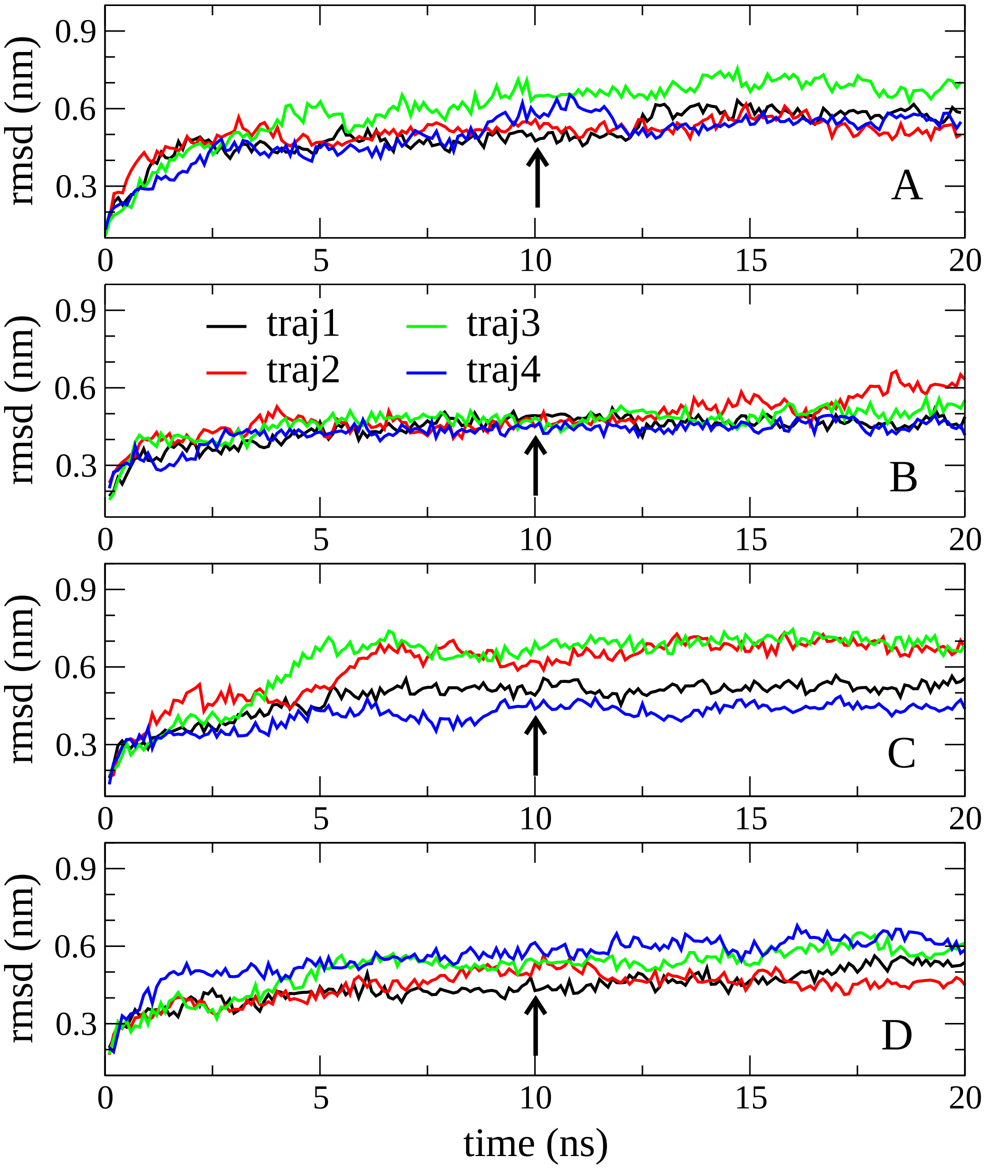

Figure S5. Time dependence of C_α RMSD of 6Aβ_{9-40}. Shown are results obtained for four independent MD trajectories in the presence of Dihydrochalcone (A), Delavinone (B), Sisalagenin (C) and Sipeimine (D). The arrow roughly refers to the time when the system reaches equilibrium.

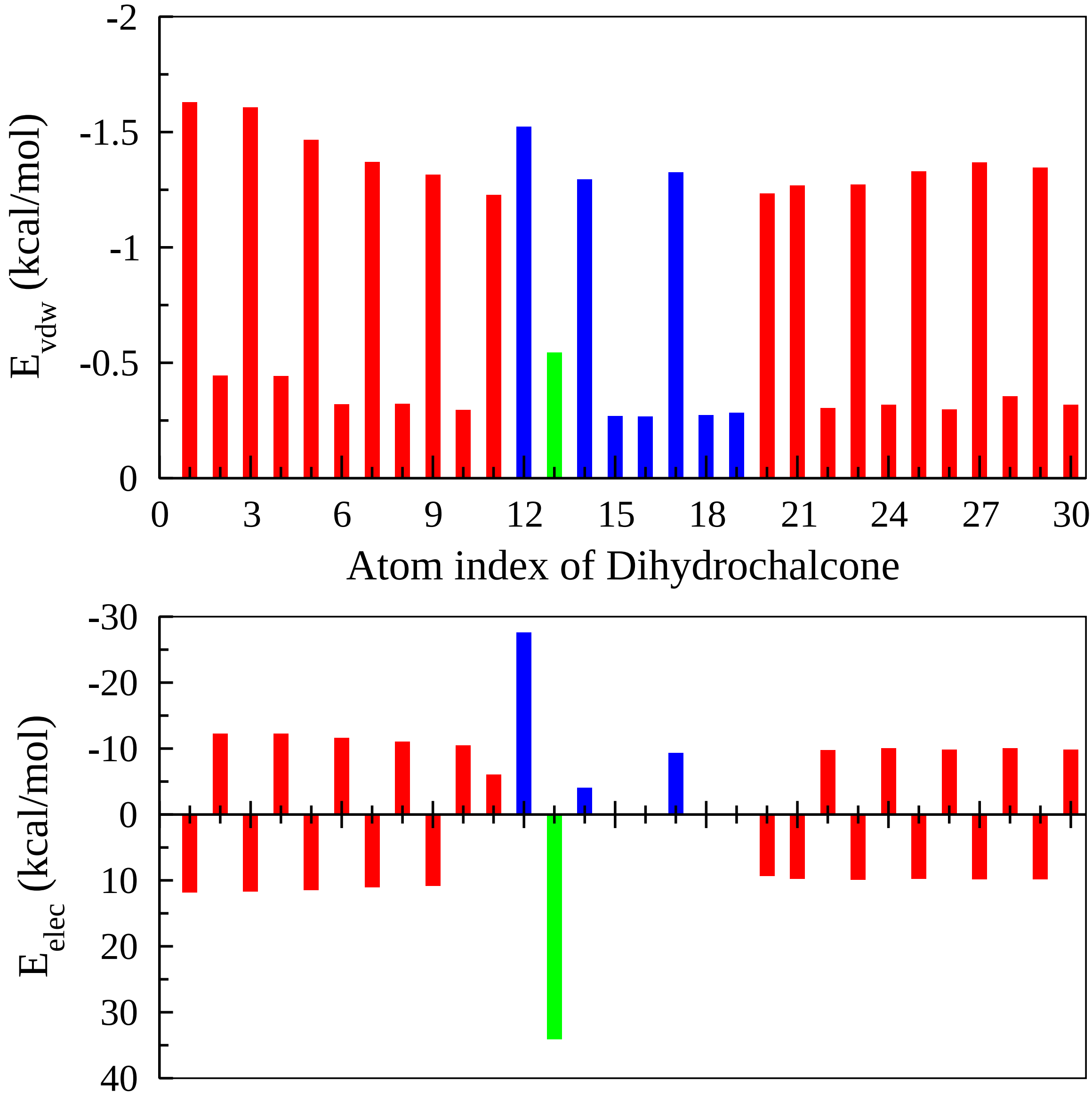

Figure S6. Contributions of individual atoms to the van der Waals and electrostatic interactions. Red refers to atoms from aromatic rings, while oxygen is denoted by green. Aother atoms between two rings are in blue.

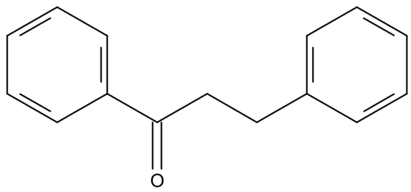

Dihydrochalcone

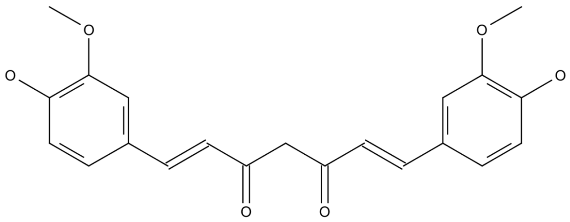

Curcumin

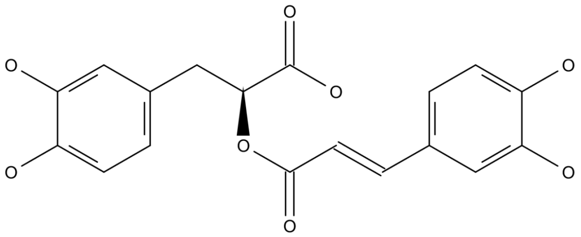

Rosmarinic acid

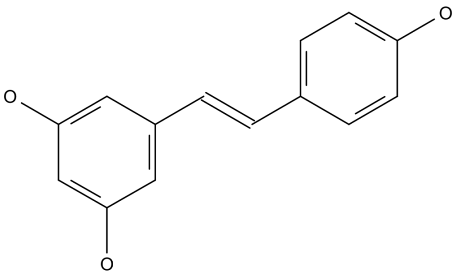

Resveratrol

Figure S7. Chemical structures of Aβ fibrillization inhibitors. From the top: Dihydrochalcone, Curcumin, Rosmarinic acid, and Resveratrol.

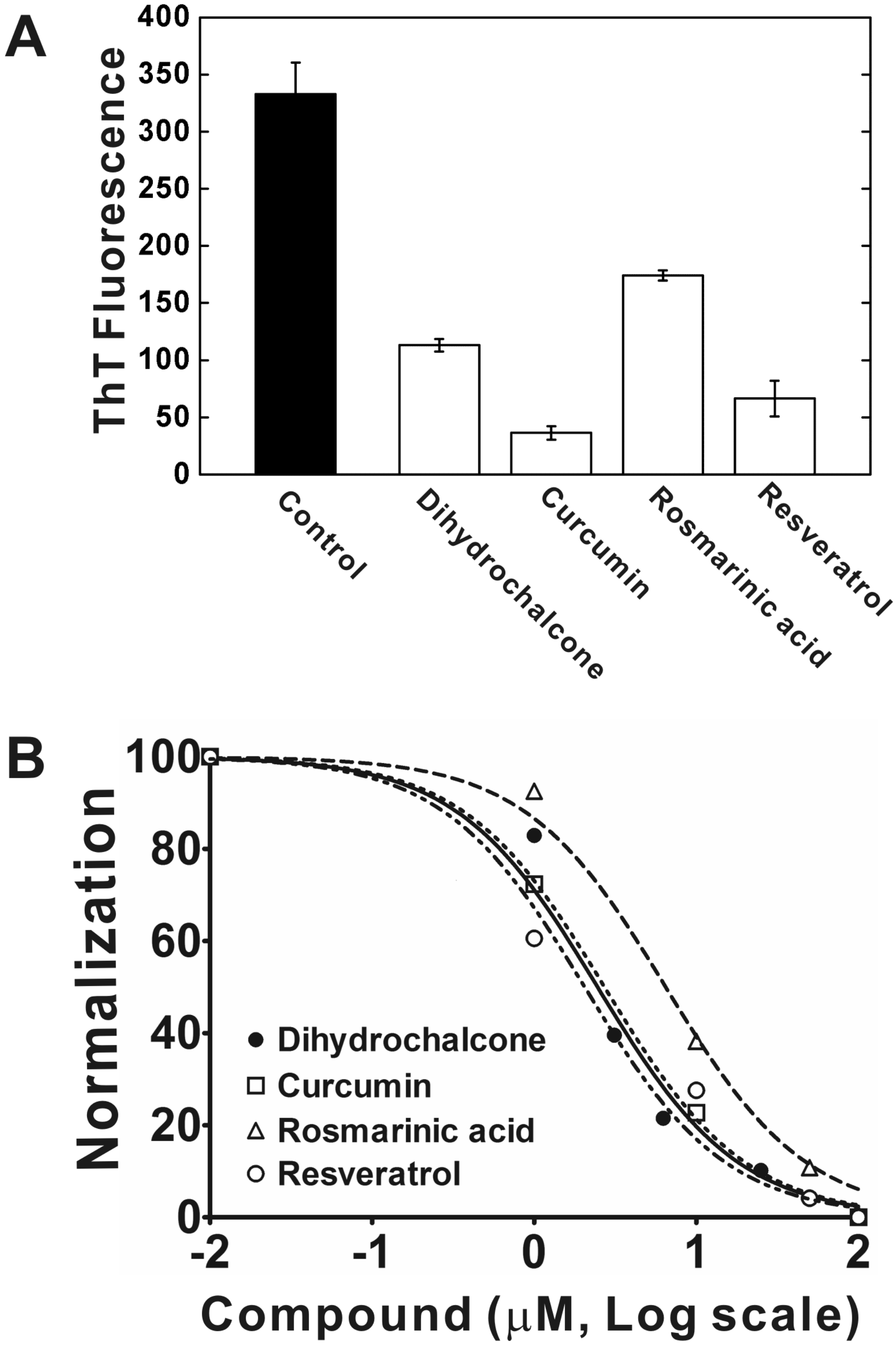

Figure S8. Aβ fibrillization in the absence and presence of Dihydrochalcone, curcumin, rosmarinic acid, and resveratrol. Aβ_{40} (25μM) was incubated at 25°C with and without 100 μM of the inhibitors and the fibrillization was monitored by ThT assay for 58 hr. The end-point ThT intensity was plotted. (B) The final ThT intensity was plotted against inhibitor concentrations and the IC50 values were calculated. Dihydrochalcone ($\bullet$), IC50 = 2.46 μM; Curcumin ($\square$), IC50=2.71 μM; Rosmarinic acid ($\triangle$), IC50=6.47 μM; Resveratrol ($\circ$), IC50 = 2.05 μM
